# An Examination of Factors Affecting Bowel Preparation for Colonoscopy: A Meta-Analysis

**DOI:** 10.1097/AJN.0000000000000246

**Published:** 2026-01-22

**Authors:** Meng Yu, Bin Cao, Hongyun Wei, Keyu Ren, Shanwei Rong, Min Li

**Affiliations:** **Meng Yu**, **Bin Cao**, **Hongyun Wei**, **Keyu Ren**, and **Shanwei Rong** are clinical nurses at the Affiliated Hospital of Qingdao University, Qingdao, Shandong Province, China, and **Min Li** is a professor at Shandong University, Jinan, Shandong Province, China. Contact author: Min Li, kyyw75@sina.com. The authors have disclosed no potential conflicts of interest, financial or otherwise.

**Keywords:** bowel preparation, colonoscopy, gastroenterology, meta-analysis

## Abstract

**Background:**

The accuracy of colonoscopy results is significantly affected by the quality of bowel preparation. Identifying and understanding the factors that contribute to effective bowel preparation are crucial for optimizing patient outcomes.

**Purpose:**

The aim of this meta-analysis was to systematically evaluate the variables influencing bowel preparation efficacy in order to inform the development of improved clinical protocols and patient care strategies.

**Methods:**

We conducted a literature search of several databases to find studies that investigated factors affecting the quality of bowel preparation over a period ranging from the inception of each database to November 5, 2024. Studies were independently screened based on preestablished inclusion and exclusion criteria. The meta-analysis was conducted using RevMan 5.4 software.

**Results:**

The meta-analysis included 27 articles containing data from 30,716 patients who underwent bowel preparation for colonoscopy. Eight factors were identified that were significantly associated with suboptimal bowel preparation: older age (odds ratio [OR], 1.28; 95% CI, 1.12-1.68; *P* = 0.019), male gender (OR, 1.33; 95% CI, 1.16-1.53; *P* < 0.001), chronic constipation (OR, 3.46; 95% CI, 2.65-4.09; *P* < 0.001), diabetes (OR, 4.72; 95% CI, 2.18-10.22; *P* < 0.001), use of opioids (OR, 1.62; 95% CI, 1.24-2.45; *P* = 0.006), a preprocedure high-fiber diet (OR, 2.94; 95% CI, 1.65-5.22; *P* < 0.001), an interval exceeding five hours between the final administration of the bowel cleansing agent and the procedure (OR, 2.82; 95% CI, 1.79-4.45; *P* < 0.001), and a last bowel movement that is not watery (OR, 4.78; 95% CI, 2.35-9.71; *P* < 0.001).

**Conclusion:**

Health care providers, including nurses, should consider these determinants of bowel preparation effectiveness and implement appropriate interventions in a timely manner to enhance patient education and care.

Good bowel preparation is a prerequisite for ensuring the quality and effectiveness of colonoscopy, which plays a crucial role in the screening, diagnosis, and treatment of colon cancer. In recent years, research on the quality of bowel preparation for colonoscopy has expanded across many regions. Guidelines have been published in Europe, the United States, and China that address bowel preparation protocols prior to colonoscopy.^1^-^3^ These guidelines serve as a benchmark for the standardization and practical application of bowel preparation in clinical settings. The process of bowel preparation for colonoscopy is complex and involves multiple components, such as assessment, bowel cleansing, diet, exercise, health education, procedures, and evaluation. Exercise is known to stimulate gastrointestinal motility, which can facilitate the clearance of the colonic contents, thereby improving visibility during colonoscopy. Studies show that engaging in moderate physical activity before colonoscopy may enhance the efficacy of bowel cleansing, potentially minimizing the reliance on supplementary measures like laxatives or enemas.^4^-^6^ However, the heterogeneity of patient populations introduces a further dimension to bowel preparation: protocols may need to be individualized to address the needs of diverse patient groups. Such individualization can involve adjustments to the duration of the preparation phase and the dosage of cleansing agents that are tailored to the unique characteristics and clinical needs of each patient.^7^,^8^

The accuracy of colonoscopy diagnosis largely depends on the quality of bowel cleansing.^9^ However, at present, the rate of satisfactory bowel preparation is estimated to be about 75%,^10^,^11^ a figure that falls short of the 90% benchmark for bowel preparation adequacy established in the recent consensus recommendations of the U.S. Multi-Society Task Force on Colorectal Cancer.^3^ To attain the quality standards recommended in the guidelines, health professionals, including nurses, should understand the factors affecting bowel preparation. This will enable them to provide effective patient education, care, and support to ensure high-quality, successful examinations.

Numerous factors may contribute to the high incidence of suboptimal bowel preparation. Patient-related factors can include noncompliance with the preparation guidelines, intricacy of the preparation regimen, and variations among individuals in gastrointestinal motility. Furthermore, the presence of comorbidities, the intake of specific medications, and patients' dietary and lifestyle habits can also influence the quality of bowel cleansing. Inadequate bowel preparation may reduce the effectiveness of detecting colorectal lesions and the success rate of cecal intubation, prolong the duration of the endoscopic procedure, and increase the risk of procedural complications.^12^ It can also lead to undergoing repeat colonoscopies, resulting in increased medical costs.^13^

Researchers have examined the factors that can impact bowel preparation quality and have identified that patients' demographic profiles, concurrent health conditions, and intake of certain medications may all play a role in shaping the effectiveness of the preparation process.^14^,^15^ However, there exists a notable degree of variability in the conclusions drawn by different studies. This inconsistency may be attributed to several methodological factors, such as limited sample size, differences in types of bowel cleansing agents, and the wide array of factors considered across studies. In light of the growing literature on this subject, the present investigation employs a meta-analytic approach to systematically synthesize and critically appraise the current evidence. This meta-analysis seeks to delineate some of the complex factors that affect bowel preparation quality, thereby providing additional evidence-based insights to inform clinical decision-making and improve patient care within the discipline of gastroenterology.

## METHODS

Our meta-analysis was conducted and is reported in accordance with the Preferred Reporting Items for Systematic Reviews and Meta-Analyses (PRISMA) guidelines.^16^ Ethical approval and patient informed consent were deemed unnecessary for this study, as it was a meta-analysis.

**Inclusion and exclusion criteria**. Articles were eligible if they met the following inclusion criteria: a cohort, case-control, or cross-sectional study; the study population comprised individuals undergoing colonoscopy, without restrictions based on age, gender, or setting (inpatient or outpatient); research focused on the analysis of factors influencing the quality of bowel preparation; and the outcome measure was based on a validated bowel preparation quality assessment scale (such as, the Boston Bowel Preparation Scale, which is widely used in clinical practice to evaluate the quality of bowel cleansing). The language of the published reports was restricted to Chinese or English. Duplicate publications, review articles or conference abstracts, and studies where the full text or complete dataset was inaccessible were excluded.

**Search strategy**. A computer-based search was conducted of the following databases: the global Cochrane Library, the U.S.-based PubMed, MEDLINE, and ClinicalTrials.gov, the European Embase, and the China National Knowledge Infrastructure, Wanfang, and Weipu databases. The aim was to collect studies on factors influencing the quality of bowel preparation; the search dates ranged from each database's inception to November 5, 2024. The search terms were determined using a combination of subject headings and free-text words, and the search strategy was as follows: *colonoscopy* AND *bowel preparation* OR *cleaning* OR *evacuation* OR *cathartics* OR *purgative* AND *risk* OR *correlation* OR *influencing* OR *factor* OR *predictor*.

**Literature screening**. Two of us independently and meticulously reviewed the literature, rigorously extracting pertinent data and cross-referencing the findings against the predefined inclusion and exclusion criteria. This dual assessment process ensured the precision and uniformity of the data collection methodology. In cases where discrepancies were identified, the issue was escalated to the full research team for consideration, where through collaborative deliberations, consensus was reached.

**Data extraction**. The scope of data extraction encompassed fundamental details about the included studies, including the principal investigator, year of publication, and geographical setting; study design and critical aspects of quality assessment; demographic characteristics of the study population; bowel preparation protocols utilized; instruments and scales employed to assess the quality of bowel preparation; variables influencing bowel preparation quality; and outcome metrics pertaining to the efficacy of bowel cleansing. Factors affecting the quality of bowel preparation were systematically identified through a comprehensive literature review, expert consultation, and by evaluating established risk factors associated with the various conditions.

**Literature quality assessment**. We used the Newcastle–Ottawa Scale (NOS) to evaluate the quality of case–control and cohort studies.^17^ The NOS assesses the quality of studies based on three categories: study group selection, study group comparability, and the determination of exposure or outcome, with a total of eight items and a maximum score of nine points. Studies are rated as low (a score of 0 to 4), medium (5 or 6), or high quality (7 to 9).^17^ For cross-sectional studies, we used the literature quality assessment checklist recommended by the Agency for Healthcare Research and Quality (AHRQ).^18^ The AHRQ checklist includes 11 items; response options for each item are “yes” (1 point), “no” (0 points), or “unclear” (0 points). Based on the total score, studies are rated low (0 to 3 points), medium (4 to 7), or high quality (8 to 11). Two of us independently conducted the quality assessment of the included studies.

**Statistical methods**. The meta-analysis was conducted using RevMan 5.4 software. A stepwise regression approach was employed to address multicollinearity in variable selection, facilitating the identification and exclusion of variables with high intercorrelations. For categorical outcomes, the odds ratio (OR) was the measure of effect size, accompanied by its corresponding 95% confidence intervals. The heterogeneity of studies was assessed; a *P* value ≥0.1 and an *I*^2^ statistic ≤50% indicated low heterogeneity, warranting the application of a fixed-effects model for pooling the data. Conversely, a *P* value <0.1 and an *I*^2^ statistic >50% indicated substantial heterogeneity, prompting sensitivity or subgroup analyses to investigate potential sources. In cases where significant heterogeneity persisted, a random-effects model was applied to the pooled analysis. Funnel plots and Egger regression tests were utilized to evaluate publication bias across risk factors. A leave-one-out sensitivity analysis was performed, sequentially omitting each study to reassess the pooled effect size and heterogeneity metrics, thereby ascertaining the impact of individual studies on the aggregate estimate. Statistical significance in this meta-analysis was set at *P* < 0.05.

## RESULTS

**Figure 1.**
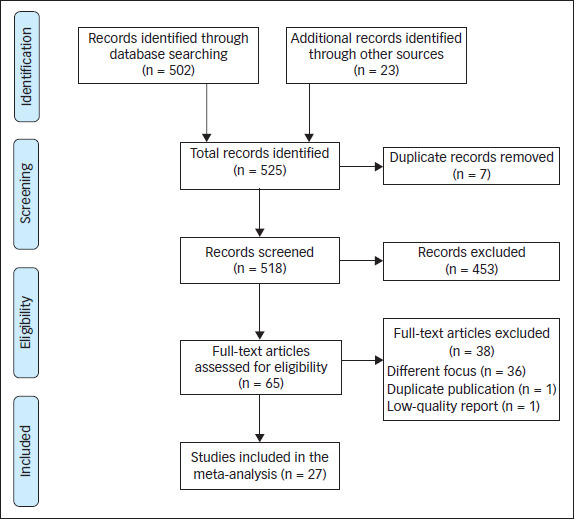
PRISMA Flow Diagram of Study Selection

**Literature search results**. The initial search yielded 525 articles. After a careful screening process, a total of 27 articles with 30,716 patients were included in the meta-analysis.^19^-^45^ See Figure 1 for the literature screening process.

**Characteristics and quality of included studies**. As delineated in Table 1,^19^-^45^ the 27 studies in this analysis were comprehensive in terms of design, including three case–control, three cohort, and 21 cross-sectional studies. Among the 30,716 total patients, 11,628 (37.9%) experienced inadequate bowel preparation. The majority of studies adhered to a similar polyethylene glycol (PEG)–based bowel preparation protocol. Most colonoscopies in the studies were scheduled in the morning, with patients following a split-dose regimen accordingly. As evidenced by the NOS scores (7 or 8), the case–control and cohort studies were of high quality (see Table 2,^26^,^31^,^32^,^36^,^38^,^39^). Similarly, the cross-sectional studies received AHRQ checklist assessment scores ranging from 7 to 9, except for one study that received a score of 6 (see Table 3,^19^-^25^,^27^-^30^,^33^-^35^,^37^,^40^-^45^).

**Table 1.** Characteristics of Included Studies

Study	Design	Country	Sample Size	Bowel Preparation Program	Assessment Tool for Bowel Preparation Quality	Influencing Factors for Poor Bowel Preparation
Anklesaria et al,^19^ 2019	Cross-sectional	United States	1,314	4 L PEG	Aronchick scale	Male gender, smoking, chronic constipation, diabetes, other comorbidities^a^
Appannagari et al,^20^ 2014	Cross-sectional	United States	3,741	4 L PEG	Aronchick scale	Older age, male gender, other sociodemographic factors,^b^ other procedure-related factors^c^
Chan et al,^21^ 2011	Cross-sectional	Malaysia	501	2 L PEG	Aronchick scale	Incomplete bowel cleansing, other sociodemographic factors,^b^ other procedure-related factors^c^
Chen et al,^22^ 2018	Cross-sectional	China	197	3 L PEG	BBPS	Older age, high-fiber diet before procedure, last bowel movement is not watery, other procedure-related factors^c^
Cheng et al,^23^ 2015	Cross-sectional	China Taiwan	703	4 L PEG	OBPS	High-fiber diet before procedure, last bowel movement is not watery
Cheng et al,^24^ 2017	Cross-sectional	China Taiwan	1,404	3 L PEG	BBPS	Older age, chronic constipation, incomplete bowel cleansing, other sociodemographic factors^b^
Chung et al,^25^ 2009	Cross-sectional	South Korea	362	4 L PEG	Aronchick scale	Older age, diabetes, history of abdominal surgery
Fang et al,^26^ 2016	Cohort	China	409	2 L PEG	BBPS	Chronic constipation, high-fiber diet before procedure, incomplete bowel cleansing
Fayad et al,^27^ 2013	Cross-sectional	United States	2,163	4 L PEG	Aronchick scale	Older age, smoking, diabetes, hypertension, other comorbidities,^a^ other sociodemographic factors^b^
Garber et al,^28^ 2019	Cross-sectional	United States	8,819	4 L PEG	Aronchick scale	Use of opioids, high-fiber diet before procedure, other procedure-related factors^c^
Ji et al,^29^ 2015	Cross-sectional	China	526	2 L PEG	OBPS	Male gender, >5-hour interval between last bowel cleansing medication and procedure, other procedure-related factors^c^
Lee et al,^30^ 2017	Cross-sectional	South Korea	404	4 L PEG	OBPS	Chronic constipation, diabetes
Li et al,^31^ 2020	Case–control	China	455	3 L PEG	BBPS	Chronic constipation, diabetes, high-fiber diet before procedure, other procedure-related factors^c^
Lim et al,^32^ 2012	Case–control	South Korea	184	4 L PEG	Aronchick scale	History of abdominal surgery
Nguyen, Wieland,^33^ 2010	Cross-sectional	United States	300	2 L PEG	Aronchick scale	Incomplete bowel cleansing, other sociodemographic factors,^b^ other procedure-related factors^c^
Paik et al,^34^ 2019	Cross-sectional	South Korea	90	2 L PEG	BBPS	Chronic constipation
Papastergiou et al,^35^ 2016	Cross-sectional	Greece	171	4 L PEG	OBPS	Male gender, >5-hour interval between last bowel cleansing medication and procedure, other procedure-related factors^c^
Rebhun et al,^36^ 2022	Cohort	United States	4,279	4 L PEG	BBPS	Male gender, other sociodemographic factors^b^
Seo et al,^37^ 2012	Cross-sectional	South Korea	366	4 L PEG	OBPS	High-fiber diet before procedure, >5-hour interval between last bowel cleansing medication and procedure
Shah et al,^38^ 2019	Case–control	United States	1,555	4 L PEG	BBPS	Smoking, diabetes, use of opioids, incomplete bowel cleansing, other comorbidities,^a^ other sociodemographic factors^b^
Sharara et al,^39^ 2016	Cohort	Lebanon	541	4 L PEG	Aronchick scale	Other sociodemographic factors^b^
Woo et al,^40^ 2018	Cross-sectional	South Korea	399	2 L PEG	Aronchick scale	Older age, history of abdominal surgery, other comorbidities^a^
Wu et al,^41^ 2011	Cross-sectional	China Taiwan	789	3 L PEG	OBPS	Older age, chronic constipation, high-fiber diet before procedure, other sociodemographic factors^b^
Wu et al,^42^ 2021	Cross-sectional	China	158	3 L PEG	BBPS	High-fiber diet before procedure, incomplete bowel cleansing, >5-hour interval between last bowel cleansing medication and procedure, last bowel movement is not watery, other procedure-related factors^c^
Xu et al,^43^ 2017	Cross-sectional	China	380	2 L PEG	BBPS	>5-hour interval between last bowel cleansing medication and procedure, last bowel movement is not watery, other procedure-related factors^c^
Yang et al,^44^ 2018	Cross-sectional	China	223	2 L PEG	BBPS	Chronic constipation, last bowel movement is not watery, other procedure-related factors^c^
Zhang et al,^45^ 2017	Cross-sectional	China	283	3 L PEG	OBPS	Older age, chronic constipation, history of abdominal surgery, incomplete bowel cleansing, last bowel movement is not watery, other procedure-related factors^c^

BBPS = Boston Bowel Preparation Scale; OBPS = Ottawa Bowel Preparation Scale; PEG = polyethylene glycol.

a Other comorbidities are coronary artery disease, cerebrovascular disease, cirrhosis, dementia, and psychiatric illness.

b Other sociodemographic factors are body mass index, obesity, low education, Medicaid, race, and single status.

c Other procedure-related factors are afternoon colonoscopy, bowel preparation intolerance, increased bowel preparation-to-defecation interval, lack of physical activity after bowel preparation, long wait time to colonoscopy appointment, and use of numerous long-term medications.

**Table 2.** The Newcastle–Ottawa Scale Score of Case–Control and Cohort Studies

Study	Patient Selection	Comparability	Exposure Assessment	NOS Total Score
Fang et al,^26^ 2016	3	2	2	7
Li et al,^31^ 2020	3	2	2	7
Lim et al,^32^ 2012	3	2	2	7
Rebhun et al,^36^ 2022	3	2	2	8
Shah et al,^38^ 2019	3	2	3	8
Sharara et al,^39^ 2016	3	2	2	7

NOS = Newcastle–Ottawa Scale.

**Table 3.** An AHRQ Quality Assessment of Cross-Sectional Studies

Study	(1)	(2)	(3)	(4)	(5)	(6)	(7)	(8)	(9)	(10)	(11)	Total Score
Anklesaria et al,^19^ 2019	Y	Y	Y	Y	Y	Y	Y	Y	N	Y	N	9
Appannagari et al,^20^ 2014	Y	Y	Y	Y	Y	Y	Y	N	N	Y	N	8
Chan et al,^21^ 2011	Y	Y	Y	Y	Y	Y	N	Y	N	N	N	7
Chen et al,^22^ 2018	Y	Y	Y	Y	Y	Y	N	Y	N	N	N	7
Cheng et al,^23^ 2015	Y	Y	Y	Y	Y	Y	Y	N	N	Y	N	8
Cheng et al,^24^ 2017	Y	Y	Y	Y	Y	Y	N	Y	N	N	N	7
Chung et al,^25^ 2009	Y	Y	Y	Y	Y	Y	N	N	N	N	N	6
Fayad et al,^27^ 2013	Y	Y	Y	Y	Y	Y	Y	N	N	Y	N	8
Garber et al,^28^ 2019	Y	Y	Y	Y	Y	Y	N	Y	N	N	N	7
Ji et al,^29^ 2015	Y	Y	Y	Y	Y	Y	Y	N	N	Y	N	8
Lee et al,^30^ 2017	Y	Y	Y	Y	Y	Y	Y	N	N	Y	N	8
Nguyen, Wieland,^33^ 2010	Y	Y	Y	Y	Y	Y	Y	Y	N	N	N	8
Paik et al,^34^ 2019	Y	Y	Y	Y	Y	Y	N	Y	N	N	N	7
Papastergiou et al,^35^ 2016	Y	Y	Y	Y	Y	Y	Y	N	N	N	N	7
Seo et al,^37^ 2012	Y	Y	Y	Y	Y	Y	N	Y	N	Y	N	8
Woo et al,^40^ 2018	Y	Y	Y	Y	Y	Y	N	N	N	Y	N	7
Wu et al,^41^ 2011	Y	Y	Y	Y	Y	Y	Y	N	N	Y	N	8
Wu et al,^42^ 2021	Y	Y	Y	Y	Y	Y	Y	Y	N	Y	N	9
Xu et al,^43^ 2017	Y	Y	Y	Y	Y	Y	N	Y	N	N	N	7
Yang et al,^44^ 2018	Y	Y	Y	Y	Y	Y	Y	N	N	N	N	7
Zhang et al,^45^ 2017	Y	Y	Y	Y	Y	Y	N	Y	N	N	N	7

AHRQ = Agency for Healthcare Research and Quality; N = no; Y = yes.

Quality assessment checklist:

Was the source of information (survey, literature review) clarified? Were the inclusion and exclusion criteria for case and control groups listed or referenced from previous publications? Was the time frame for patient identification provided? If not derived from a population, were the study subjects consecutive? Did the evaluator's subjective factors overshadow other aspects of the study subjects? Were descriptions provided for any assessments conducted to ensure quality (such as, testing/retesting of primary outcome measures)? Were any reasons given for the exclusion of patients from the analysis? Were descriptions provided for measures to evaluate and/or control for confounding factors? If possible, was an explanation offered for how missing data were handled in the analysis? Was there a summary of patient response rates and the completeness of data collection? If there was follow-up, was there an identification of the percentage of expected incomplete patient data or follow-up outcomes?

**Meta-analysis**. As presented in Table 4, independent factors significantly affecting the quality of bowel preparation for colonoscopy were the following: older age (≥ 65 years) (OR, 1.28; 95% CI, 1.12-1.68; *P* = 0.019), male gender (OR, 1.33; 95% CI, 1.16-1.53; *P* < 0.001), chronic constipation (typically determined through a combination of patient-reported symptoms as noted in a stool diary and medical evaluations such as stool tests) (OR, 3.46; 95% CI, 2.65-4.09; *P* < 0.001), diabetes (OR, 4.72; 95% CI, 2.18-10.22; *P* < 0.001), use of opioids (OR, 1.62; 95% CI, 1.24-2.45; *P* = 0.006), a preprocedure high-fiber diet (determined by 24-hour dietary recall, in which individuals were asked to recall and report all the foods and beverages they consumed over the previous 24 hours, which could be analyzed for fiber content) (OR, 2.94; 95% CI, 1.65-5.22; *P* < 0.001), an interval exceeding five hours between final administration of the bowel cleansing agent and the procedure (OR, 2.82; 95% CI, 1.79-4.45; *P* < 0.001), and a last bowel movement that is not watery (OR, 4.78; 95% CI, 2.35-9.71; *P* < 0.001).

**Table 4.** Meta-Analysis of Factors Affecting the Quality of Bowel Preparation for Colonoscopy

		Heterogeneity			Synthesized Outcomes		
Factors	No. of Included Studies^a^	*I* 2	*P*	Effect Model for Analysis	OR	95% CI	*P*
Older age	6	55	0.01	Random	1.28	1.12-1.68	0.019
Male gender	6	63	0.02	Random	1.33	1.16-1.53	<0.001
BMI	4	24	0.14	Fixed	1.22	1.05-1.44	0.051
Smoking	2	93	<0.01	Random	1.75	0.85-3.58	0.138
Chronic constipation	7	76	<0.01	Random	3.46	2.65-4.09	<0.001
Diabetes	5	94	<0.01	Random	4.72	2.18-10.22	<0.001
Hypertension	3	87	<0.01	Random	2.14	0.87-3.18	0.106
History of abdominal surgery	3	96	<0.01	Random	1.57	0.23-0.75	0.642
Use of opioids	3	77	0.05	Random	1.62	1.24-2.45	0.006
High-fiber diet before procedure	6	92	<0.01	Random	2.94	1.65-5.22	<0.001
>5-hour interval between final administration of bowel cleansing agent and the procedure	2	0	0.93	Fixed	2.82	1.79-4.45	<0.001
Last bowel movement is not watery	6	83	<0.01	Random	4.78	2.35-9.71	<0.001

BMI = body mass index; OR = odds ratio.

a The number of studies may not match the number listed in Table 1. This is because certain study data were not amenable to extraction for the meta-analysis (for reasons having to do with reporting of effect sizes and variance).

**Sensitivity analysis**. We performed an analysis of the influential factors included in the study using both fixed-effects and random-effects models, yielding essentially congruent results. Furthermore, by evaluating the impact of a sequential exclusion of each study on the overall findings, we observed minimal significant statistical variation in the outcomes. This observation suggests that the results of the meta-analysis exhibit a high degree of stability.

**Publication bias**. Given that the number of studies in each outcome analysis was less than 10, we were precluded from constructing funnel plots to evaluate publication bias. However, the results of Egger regression analysis revealed no evidence of significant publication bias across the studies, with all *P* values exceeding the threshold of 0.05.

## DISCUSSION

The ideal bowel preparation for a colonoscopy should reliably and quickly clear all fecal matter from the colon without causing significant histological changes to the colonic mucosa.^46^ Inadequate bowel preparation can markedly compromise the diagnostic efficacy of colonoscopy by diminishing visibility of the colonic mucosa, potentially leading to missed lesion detection and affecting the accuracy of disease diagnosis. Additionally, inadequate preparation may prolong the procedural time required for cecal intubation, negatively impact the success rate of achieving complete cecal intubation, lead to more frequent colonoscopies, and ultimately increase overall health care costs.^47^,^48^

The results of this meta-analysis show that advanced age, male gender, chronic constipation, diabetes mellitus, opioid use, a high-fiber diet prior to the procedure, a delay exceeding five hours between the final administration of the bowel cleansing agent and the procedure, and a nonwatery consistency of the most recent bowel movement are independent factors that significantly impact the quality of bowel preparation for colonoscopy. The broad spectrum of influencing factors identified in this study, and in another meta-analysis published after our study was concluded,^49^ may furnish a more robust and scientifically grounded evidence base, which can inform the development of targeted intervention strategies to ensure that patients are adequately prepared for colonoscopy and to optimize outcomes.^50^

In keeping with previous investigations, we found that age is a significant factor influencing the quality of bowel preparation. According to one study conducted among hospitalized patients, for every increase of 10 years in age, the odds of having inadequate bowel preparation increased by 1.29.^51^ PEG bowel preparation for colonoscopy necessitates the ingestion of substantial fluid volumes, which may pose a challenge for older adults. Older patients may have a reduced tolerance to high fluid intake, which can lead to adverse reactions such as nausea and vomiting, resulting in reduced compliance. Additionally, a decrease in daily physical activity due to reduced mobility in the elderly can lead to reduced bowel peristalsis, which may contribute to suboptimal bowel preparation quality.^6^ A recent study on exercise found an association between physical activity and the effectiveness of bowel cleansing.^48^ However, the literature does not provide conclusive guidelines for the optimal amount or intensity of physical activity, especially for older adults, during the bowel preparation period. This knowledge gap highlights the necessity for additional research to formulate evidence-based recommendations for clinical practice.

We also found that the quality of bowel preparation of male patients was lower than that of female patients, which is consistent with previous studies.^52^ This finding may be related to lower compliance among men.^53^ However, a smaller number of studies have found no significant gender difference when it comes to the quality of bowel preparation.^54^,^55^ It may be that the variation in research findings regarding gender reflects cultural factors, differences in education, and the exclusion of noncompliant patients. Consequently, the role of gender in determining bowel preparation quality merits further exploration.

Chronic constipation, which impacts bowel preparation, is associated with the presence of comorbidities such as diabetes mellitus and/or stroke, the consumption of opioid medications, physical activity levels, and dietary habits, among others. Individuals with chronic constipation frequently exhibit a decline in autonomic nervous system function and intestinal muscle activity, resulting in reduced intestinal motility and prolonged transit time for bowel movements.^56^ This results in a large amount of fecal residue within the intestinal lumen, which affects the quality of bowel preparation. Patients may need to take a higher dose of the prescribed laxative or use an enema.^57^

Our meta-analysis shows that diabetes mellitus was associated with poor bowel preparation quality. Previous studies indicate that, relative to healthy individuals, patients with diabetes exhibit reduced intestinal motility and experience significantly elongated defecation intervals.^58^,^59^ Additional challenges faced by patients with diabetes that could impact bowel preparation include gastroparesis, autonomic neuropathy, fluid–electrolyte imbalances, and complications related to adjustments to antihyperglycemic medications and dietary restrictions prior to colonoscopy.^60^,^61^ It is imperative for health care professionals to vigilantly monitor patients for signs of hyperglycemia, and to maintain blood glucose levels within normal parameters. However, as with exercise recommendations, adequate guidelines on specific bowel preparation strategies for patients with diabetes are lacking, and additional research is needed.^60^,^61^

Opioids are utilized across a spectrum of medical contexts, primarily for the management of moderate to severe pain. The dosage and regimen of opioids are tailored to the individual; for example, treatment is generally started at a low dose, with subsequent modifications based on the patient's response and tolerability. The objective is to achieve optimal analgesia while minimizing adverse effects. These medications exert a range of modulatory actions on the intestinal nervous system through the activation of opioid receptors, leading to a reduction in propulsive peristalsis and an increase in tone of the gastrointestinal sphincters. This results in delayed intestinal transit and enhanced fluid absorption, predisposing patients to constipation and, by extension, compromising the quality of bowel preparation. It is not always possible to restrict opiates in the bowel preparation of patients with severe pain.^28^ However, in clinical practice, the use of opioid medications and the frequency of bowel movements should be closely scrutinized to determine if adjustments can be made. These may include a longer preparation period, lifestyle changes, and the use of additional laxatives.

A diet abundant in fiber may extend the intervals between bowel movements, leading to significant fecal accumulation in the intestinal tract and consequently complicating the bowel cleansing process. However, criteria defining the amount of fiber in a diet are not uniform, calling for further elucidation. Previous research has shown that the fiber content in a patient's diet is inversely correlated with the efficacy of bowel cleansing.^9^ Relevant guidelines and studies^1^,^10^,^62^ recommend adopting a low-fiber diet prior to colonoscopy, with dietary restrictions typically not exceeding 24 hours.^7^

As reflected in the literature, we found that a prolonged interval between the last dose of a bowel cleansing agent and the time of the procedure can impact the quality of colonic cleansing, particularly on the right side of the colon.^9^ Longer intervals can lead to a continuous influx of intestinal fluid or fecal matter from the small intestine into the colon. It is recommended that the interval between the final administration of a bowel cleansing agent and the commencement of the colonoscopy should ideally be four hours or less, and under normal circumstances should not exceed seven hours, to ensure the optimal effectiveness of bowel cleansing before the procedure.^1^,^35^

The characteristics of the last bowel movement can reflect the status of intestinal evacuation and can be used clinically to assist medical staff in predicting the quality of bowel preparation. According to a study by Fatima and colleagues, among patients who described their last bowel movement as brown liquid or containing solid stool, 54% had inadequate bowel preparation quality.^63^ It is suggested that patients whose last stool is not watery be given additional laxatives or undergo remedial measures such as enemas.

As consensus gradually forms regarding improved regimens and strategies for bowel preparation, researchers are also focusing on patient health education and the use of technology to achieve better results. At present, preprocedural bowel preparation education is predominantly delivered by nursing staff. In recent years, there has been a notable proliferation of interventional studies focusing on educational guidance, which offers valuable insights.^64^ Investigators have employed virtual reality technology and utilized educational video clips sent via smartphone to familiarize patients with the bowel cleansing and colonoscopy processes, enhancing patient understanding and compliance.^65^-^67^ One study reported that the use of personalized smartphone applications significantly improved bowel preparation quality and increased the detection rate of polyps in the right colon.^68^ Currently, interventions that integrate multimedia and internet technologies to augment the quality of bowel preparation have garnered considerable attention from the academic community, and health professionals are poised to delve deeper into innovative research pathways within the bowel preparation process for colonoscopy. This exploration will likely encompass the integration of intelligent devices, patient health education initiatives, and the refinement of procedural protocols to enhance the patient experience and overall efficacy of bowel preparation.

**Limitations**. The limitations of this study are multifaceted and require careful consideration. First, the studies included in our analysis applied diverse definitions and reporting methodologies for certain influencing factors. For example, the scales used to assess bowel cleansing varied, and variables such as age and body mass index were treated as continuous in some studies and as categorical in others. This lack of uniformity complicates the execution of a comprehensive, integrated data analysis. Second, the studies encompassed a broad spectrum of ethnicities, nationalities, and geographic regions, which, when coupled with inconsistent definitions of bowel preparation quality and unclear reporting of certain factors like diabetes and opioid use, may introduce heterogeneity that affects the generalizability of our findings. Third, most of the studies were cross-sectional, which inherently restricts our ability to establish causal relationships and could impact the reliability of our conclusions. It is recognized that the variables considered as risks may vary among studies due to differences in study populations, research objectives, and specific outcomes of interest. Our selection process aimed to identify variables most pertinent and significant to our study's context, ensuring transparency and replicability for future research. Finally, our review was limited to literature published in Chinese and English, potentially excluding studies in other languages and introducing a bias toward the identified literature. These limitations should be considered when interpreting the results of this study and when contemplating future research directions in the field of bowel preparation for colonoscopy.

## CONCLUSIONS

In clinical practice, health care providers should perform a thorough evaluation of patient health conditions, considering the factors identified in our study, in order to enhance the quality of bowel preparation. Furthermore, the incorporation of intelligent devices, targeted patient health education, and the optimization of bowel preparation protocols are expected to be key areas of future research. These developments are poised to foster innovation and enhance clinical outcomes in the area of colonoscopy procedures.
